# Mindfulness Intervention Benefits Older Adults Receiving Rehabilitation Services in Long Term Care

**DOI:** 10.1093/geroni/igaa057.3361

**Published:** 2020-12-16

**Authors:** Katarina Felsted, Katherine Supiano, Trinh Mai, Anthony Muradas

**Affiliations:** University of Utah, Salt Lake City, Utah, United States

## Abstract

Research literature includes preliminary examination of mindfulness in rehabilitation settings; however, further investigation is warranted. Some of the strongest findings to date are adaptation improvements such as self-efficacy, increased quality of life, and decreased stress. The purpose and aims of this pilot feasibility and acceptability study were to develop, administer, and evaluate a modified mindfulness program for older adults in rehabilitation in long term care, and to measure self-efficacy, quality of life, and perceived stress. Nine residents 65+ were recruited. Inclusion criteria for participants included residents receiving any type of therapy (e.g., physical, occupational, speech), an anticipated length of stay inclusive of the intervention treatment period, and cognitive capacity to participate. A mindfulness intervention was developed by the research team and administered by a CITI trained, qualified mindfulness instructor. As this is a pilot study, no control group was used. This study proved both feasible and acceptable. All eligible participants consented; both attendance and retention percentages were above the 75% standard (78% and 89%, respectively), and the Meaningful Activities Scale rating=82.4, indicating strong acceptability. Statistical results values for the Health-Related Quality of Life (V=153, p< 0.001), Bandura’s Self Efficacy Questionnaire (V=153, p< 0.001), and Cohen’s Perceived Stress Scale (V=152, p< 0.001) were all statistically significant. These preliminary research findings will inform a larger pragmatic trial testing preliminary effectiveness of the intervention in this population in quality of life, self-efficacy and stress reduction. While this study began prior to the COVID-19 pandemic, its findings are now even more relevant to gerontology.

